# Identifying the active components through the behaviour change techniques taxonomy in complex interventions for people living with multiple long‐term health conditions: A systematic review

**DOI:** 10.1111/bjhp.70019

**Published:** 2025-08-27

**Authors:** Tasmin A. Rookes, Danielle Nimmons, Rachael Frost, Megan Armstrong, Wing Nga Tsang, Laura Davies, Jamie Ross, Jane Hopkins, Manoj Mistry, Stephanie J. C. Taylor, Kate Walters

**Affiliations:** ^1^ Department of Primary Care and Population Health UCL London UK; ^2^ Public and Allied Health Liverpool John Moores University Liverpool UK; ^3^ Wolfson Institute of Population Health QMUL London UK; ^4^ Public Contributor

**Keywords:** behaviour change techniques, complex interventions, randomized controlled trials, systematic review

## Abstract

**Background:**

More older adults are living with multiple long‐term conditions (M‐LTCs). Understanding the ‘active components’ of complex interventions to manage these is challenging. The Behaviour Change Techniques Taxonomy (BCTTv1) helps identify the ‘active components’ to understand which are associated with improved outcomes. This is important for people with M‐LTCs due to health complications, competing health care regimes and conflicting clinical teams, requiring complex decision‐making.

**Objectives:**

This systematic review explores which BCTs are associated with effective complex interventions in people with M‐LTCs.

**Methods:**

Five databases were systematically searched to identify RCTs evaluating behaviour change intervention effectiveness in people with M‐LTCs, published between 1999 and 2025. Data on intervention characteristics, effectiveness and BCTs were synthesized. A BCT index of potential was calculated by determining the percentage of studies that used a BCT that had a positive primary outcome. To be considered to have potential, a BCT had to have an index of potential higher than 50%.

**Results:**

Fifty‐nine eligible articles were included. 44/93 possible BCTs were identified, ranging from 1 to 16 different techniques per intervention (mean = 7). Thirty‐two BCTs were present in three or more studies, of which 17 had the potential to improve outcomes, such as behavioural goal setting, monitoring outcomes, problem solving and providing information about health and emotional consequences. Interventions designed for people with both physical and mental LTCs were more likely to contain BCTs with higher potential.

**Conclusions:**

Interventions delivered to those living with M‐LTCs should incorporate relevant BCTs with a high index of potential and use mechanisms of action to identify other BCTs to include alongside these.

## INTRODUCTION

Long‐term conditions (LTCs) are generally defined as a condition lasting for longer than one year, which often cannot be cured (National Institute for Health and Care Excellence, 2015); examples include diabetes, cardiovascular disease, stroke, cancer, depression, arthritis and anxiety. These conditions often do not occur in isolation, with many people living with multiple long‐term conditions (M‐LTCs), commonly defined as having two or more LTCs at the same time. The prevalence of living with M‐LTCs is reported to be 23.2% of all adults, rising to 64.9% in those aged between 65 and 84 and 81.5% in those 85 and over (Barnett et al., 2012), which is predicted to rise with an ageing population (Kingston et al., 2018). This increased prevalence puts pressure on health care services, people living with the conditions and their carers, making managing M‐LTCs a priority (Afshar et al., 2015; Barnett et al., 2012; Salisbury et al., 2011). This has been reflected through identifying effective interventions for people with M‐LTCs being set as a main priority by the James Lind Alliance, the NIHR M‐LTCs Strategic Framework and the Academy of Medical Sciences (Jame Lind Alliance, 2024; National Institute for Health and Care Research, 2024; The Academy of Medical Sciences 2018).

To enable people living with LTCs to manage their health and improve their outcomes, complex behaviour change interventions have been successfully designed and delivered (Cutler et al., 2018; Lorig et al., 2016; Warner et al., 2015). They contain multiple interacting components, which are adapted to the context and rely on the behaviours of those delivering and receiving the intervention (Skivington et al., 2021). These components may include education, physical activity, disease monitoring and/or medication review.

The complex nature of these interventions can make it difficult to define and standardize the ‘active components’ to bring about change, especially when they are often not measured or reported (Datta & Petticrew, 2013; Michie & Johnston, 2012). Not reporting or specifying these ‘active components’ is associated with smaller effect sizes of interventions (Taylor et al., 2012). To enable these ‘active components’ to be systematically categorized and compared across interventions, Abraham and Michie (2008) developed the Behaviour Change Techniques Taxonomy (BCTTv1), which consists of 93 specific techniques, grouped into 16 broader categories. This taxonomy has successfully been applied to a range of LTCs to change health behaviours, such as physical activity, healthy eating, smoking cessation and diabetes management (Gardner et al., 2017; Michie et al., 2011; Presseau et al., 2015). However, in a meta‐analysis looking at physical activity behaviour change interventions for workers, there was no evidence for specific BCTs or the number of BCTs being associated with more beneficial outcomes (Taylor et al., 2012). These findings highlight the need to understand if and how specific BCTs impact outcomes in different populations.

When developing complex behaviour change interventions for people living with M‐LTCs, evidence from interventions targeted at people with single LTCs has typically been applied (Harrison et al., 2012; Zora et al., 2021). However, the often‐fragmented care across multiple health care teams and the complex priority setting and decision‐making for people with M‐LTCs, highlights their specific care needs (Bratzke et al., 2015; Smith et al., 2012, 2016). Developing complex behaviour change interventions for people with M‐LTCs may require different approaches than those with single LTCs. Therefore, we must identify which ‘active components’ meet the needs of people living with M‐LTCs (Parker et al., 2019). A sister paper to this review found that behaviour change interventions targeted at people with M‐LTCs showed effects on only a few outcomes, mainly in relation to mental health outcomes like psychological distress (depression) and emotional wellbeing, with some subgroup analyses indicating benefits of interventions lasting for longer than 6 months (Rookes et al., 2024). Given the limited evidence of behaviour change interventions for improving the outcomes for people living with M‐LTCs, it is important to understand which behaviour change components contribute to more effective interventions to support researchers and health and care services to develop and deliver better interventions.

Historically, there had been insufficient application of theory and frameworks (like the BCTTv1) to guide complex intervention development for people with M‐LTCs, which makes learning from and building on top of previous interventions challenging (Smith et al., 2012). In recent reviews, the components important for interventions for people with M‐LTCs have been explored, with Smith et al. (2016) finding those targeted at the organizational level, those focussed on specific risk factors and those prioritizing problems patients were facing were more effective. In a more recent realist review, Kastner et al. (2019) found that the underlying mechanisms for success in chronic disease management interventions were care coordination, disease prioritization and patient self‐management. Frost et al. (2020) also found overlapping components and outcome measures were key, despite different underlying conditions, suggesting a move towards integrated care for M‐LTCs may be the best approach to improve outcomes. To date, no review has identified the active behaviour change techniques (BCTs) in complex interventions for those with M‐LTCs that are more likely to be associated with effective interventions and beneficial patient outcomes. This review was embedded within a wider review looking at the effectiveness of behaviour change interventions for people with M‐LTCs (Rookes et al., 2024).

## AIM

To identify the active behaviour change components that are associated with effective complex interventions for people with M‐LTCs.

## METHODS

This systematic review follows the PRISMA reporting guidelines (Table S1) and is registered on PROSPERO (ID: CRD42021287847). The full methods of the systematic review are published in a sister paper (Rookes et al., 2024). A summary of the methods used in both reviews is outlined in Table 1.

**TABLE 1:** Methods used in this review, full details of which can be found in sister paper (Rookes et al., 2024).

Search	Systematically searched MEDLINE, Embase, PsychInfo, CINAHL and Web of Science (Figure S1) between January 1999 (date of the oldest paper identified in a Cochrane review for complex interventions in people with M‐LTCs (Smith et al., 2016)) until January 2022. This search was updated in February 2025, identifying six additional papers. Search terms focussed on M‐LTCs, complex behaviour change interventions and randomized controlled trials and were adapted from Smith et al. (2021) and Gardner et al. (2017). 10% of titles and abstracts and 20% of full texts were independently screened by two reviewers, using Rayyan (Ouzzani et al., 2016). As there was less than 5% conflict, the remaining abstracts and full texts were single screened by one reviewer.
Inclusion criteria	Participants were adults with two or more LTCs, e.g., diabetes, stroke, depression, coronary heart disease, asthma, anxiety, arthritis, psychosis, liver disease, hypertension, some cancer, chronic pain, HIV and COPD. Complex behaviour change interventions for the management of LTCs, at individual or organizational level, delivered in primary and community care settings. Randomized controlled trials, comparing the intervention to treatment as usual. Studies were not excluded based on the primary outcome chosen.
Data extraction	Study characteristics, such as, trial author, year of publication, country, intervention description and length, follow‐up, M‐LTCs included, sample size, primary outcome and effectiveness, and behavioural target were extracted by one reviewer (TAR) and checked by another (DN or WNT). For BCTs, following BCT training (Michie et al., 2013), two reviewers independently extracted the BCTs present in each intervention (TAR for all, DN for the first search and WNT for updated search), which were then compared and discussed until consensus was agreed. BCTs were coded in relation to the person for whom the primary outcome was collected but could have been for any behavioural target.
Study quality assessment	Study quality of the eligible trials was assessed for the sister review paper using the Risk of Bias 2 (RoB2) tool (Sterne et al., 2019). Risk of bias was assessed independently by two reviewers for 20% of studies, which was extended to an additional 10% due to lack of consensus. One reviewer then assessed the bias in the remaining studies. This was used to interpret how confident we can be in the results, but no studies were excluded based on quality assessment.
Data analysis	Results from initial and updated searches were synthesized together. A pragmatic method for exploring the potential effectiveness of BCT has previously been conducted in interventions for older frail adults (Gardner et al., 2017). Following this methodology, BCTs identified in three or more studies were synthesized to identify an ‘index of potential’ for effectiveness. If more than 50% of the interventions that incorporated a BCT were found to have a positive and significant primary outcome effect, then the BCT was deemed as a potentially effective ‘active component’ for people with M‐LTCs. The sister review (Rookes et al., 2024) found that complex interventions for people with physical and mental LTCs effectively reduced psychological distress, but not for those with two or more physical LTCs. An exploratory synthesis of effective BCTs between these two populations was therefore conducted.

## RESULTS

**FIGURE 1:**
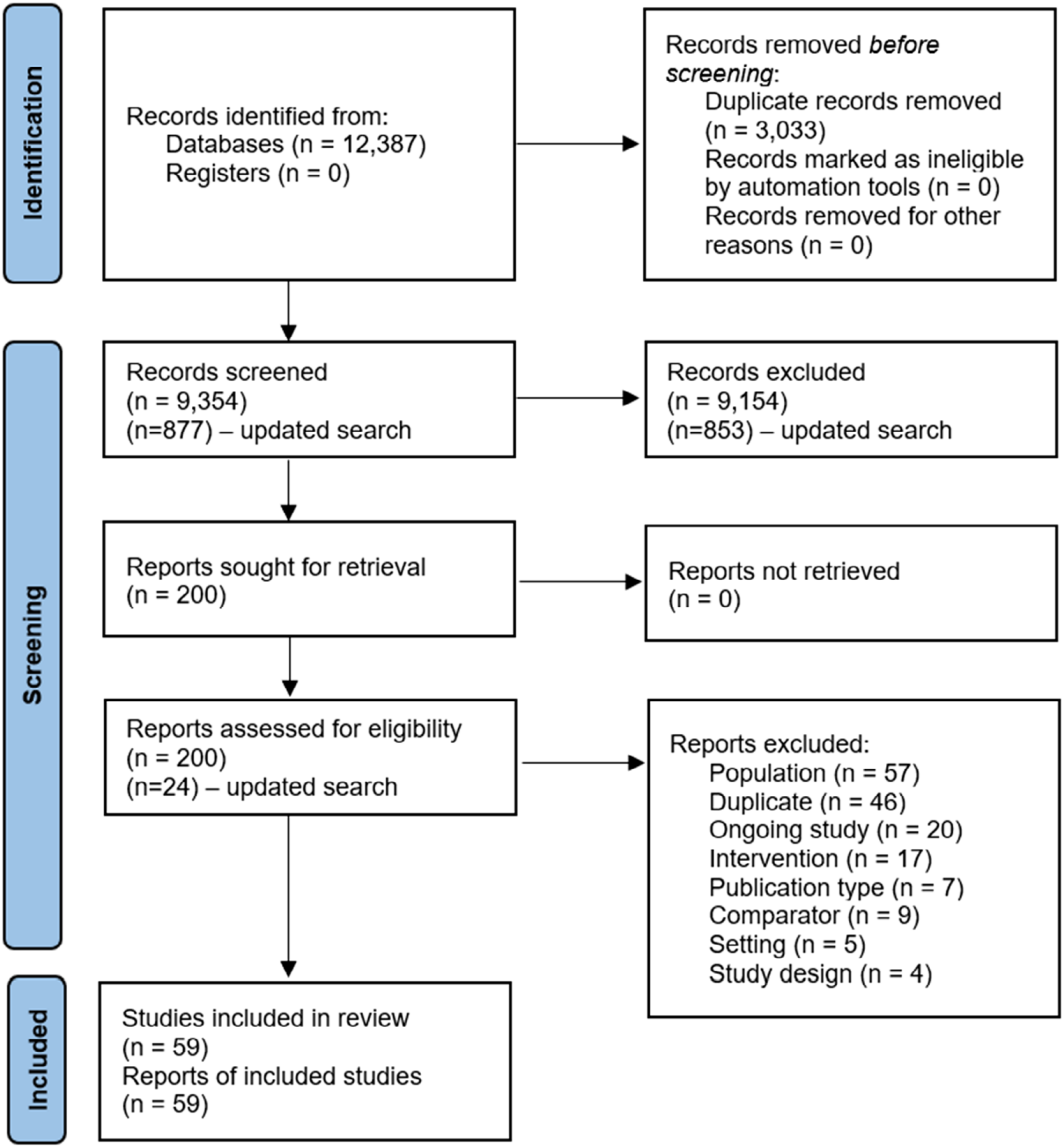
Prisma flow chart (Page et al., 2021) adapted from Rookes et al. (2024).

9354 titles and abstracts and 200 full texts were screened. For the updated search, 877 titles and abstracts and 24 full texts were screened. Fifty‐nine eligible articles were included in the synthesis (see Figures 1 and S2).

### Study characteristics

Across 59 Randomized Controlled Trials (RCTs), there were a total of 21,226 participants ranging from 25 to 3324. Full details of the included interventions from the initial search are available in the sister paper to this, and all results are summarized in Table S2 (Rookes et al., 2024). Briefly, studies were conducted in 16 countries, and results were published between 2001 and 2025. Interventions were categorized into three groups: self‐management (*n* = 30), collaborative care (*n* = 18) and cognitive and/or behavioural activation (*n* = 11) and ranged in length from 3 weeks to 2 years. They targeted participants with a variety of physical and mental health conditions, such as diabetes (*n* = 41), depression (*n* = 33), arthritis (*n* = 10), psychosis (*n* = 5) and coronary heart disease (*n* = 9).

### Intervention effectiveness

Of the 59 included studies, 56 reported the statistical outcome of the study, with 32 being effective and 24 showing no statistical benefit on the primary outcome. Psychological distress as an outcome was the most amenable to change, with 12/14 (86%) of those measuring it detecting a significant benefit to participants. Interventions with a collaborative care approach resulted in improvements across a range of outcomes, and cognitive and/or behavioural activation interventions and those targeted at people with a physical and mental LTC were beneficial for psychological distress outcomes.

Study quality was variable, with 30 studies rated as low risk of bias, 22 rated as having some concerns and seven studies assessed as having a high risk of bias (Table S2). The main issues were participants knowing which group they were in, due to the nature of behavioural interventions, and some concerns around whether this impacted outcomes. Other concerns included missing data, as many participants were lost to follow‐up in some studies, and it was unclear if these participants differed from those who remained. This is unlikely to impact the BCTs themselves but have an impact on the effectiveness of the intervention. Full results and meta‐analysis of the 53 studies identified in the initial search can be found in the sister paper to this review (Rookes et al., 2024).

### Behaviour change techniques (BCTs)

Of the 93 individual BCTs, 44 were identified as present in one or more of the 59 study interventions (Table S3). The number of BCTs within each intervention ranged from 1–16, with a mean of 7 BCTs per intervention. Examples of how these BCTs were operationalized within interventions can be seen in Table 2.

**TABLE 2:** Practical examples of how some BCTs can be operationalized within complex interventions.

BCT	Example
1.1 Goal setting (Behaviour)	Agree a daily walking goal, e.g., 3 miles
1.2 Problem solving	Identify barriers to starting a new exercise program and discuss ways to overcome them
1.3 Goal setting (Outcome)	Set a weight loss goal as an outcome of changed eating patterns
1.4 Action planning	Plan to run at certain times of the day on certain days of the week
2.3 Self‐monitoring of behaviour	Record how many steps they have done, or number of fruits and vegetables eaten in a day
2.4 Self‐monitoring of outcome(s) of behaviour	Weigh self everyday and record weight over a period of time and plot on a graph
2.5 Monitoring of outcome(s) of behaviour without feedback	Health care professional records blood pressure over time
2.6 Biofeedback	Provide feedback to person of their clinical measures to improve health behaviours
3.1 Social support (unspecified)	Support and information from an external person to help with target behaviour
5.1 Information about health consequences	Information about how not stopping smoking will impact future health
6.1 Demonstration of the behaviour	Showing people how to measure their own blood glucose levels when they will be self‐monitoring them
7.1 Prompts/cues	Alerts on health care professional's computer systems when clinical measures go outside of recommended ranges
11.1 Pharmacological support	Providing antidepressants alongside psychological support to help reduce depression symptoms

Studies most commonly had at least one BCT in the Goals and Planning (54/59, 92%) and Feedback and Monitoring categories (52/59, 88%). Within these categories, 1.2 Problem solving (*n* = 34) and 1.4 Action planning (*n* = 28) were used the most. Thirty‐three studies included some form of Goal setting (1.1—behaviour and 1.3—outcome), with two of these studies including both. For the Feedback and Monitoring category, 2.5 Monitoring of outcome(s) of behaviour without feedback was used the most (*n* = 27), followed by 2.6 Biofeedback in 18 studies. Self‐monitoring was seen in 24 studies (2.3—behaviour and 2.4—outcome(s) of behaviour), with one of these studies including both. Thirty‐two BCTs were identified in at least three studies (Table 3).

**TABLE 3:** Index of potential, exploring the potential effectiveness of the BCTs used in three or more studies.

Behaviour change technique	Number of eligible studies using technique	Number of studies using technique with an effective primary outcome	Index of potential^a^
3.2 Social support (practical)	6	0	0
15.1 Verbal persuasion about capability	5	1	20
1.5 Review behaviour goals(s)	6	2	33
2.2 Feedback on behaviour	6	2	33
3.3 Social support (emotional)	3	1	33
6.2 Social comparison	3	1	33
8.3 Habit formation	3	1	33
2.6 Biofeedback	18	8	44
1.3 Goal setting (outcome)	13	6	46
6.1 Demonstration of the behaviour	13	6	46
7.1 Prompts/cues	15	7	47
1.4 Action planning	26	13	50
2.3 Self‐monitoring of behaviour	14	7	50
2.4 Self‐monitoring of outcome(s) of behaviour	10	5	50
8.1 Behavioural practice/rehearsal	10	5	50
11.2 Reduce negative emotions	11	6	55
4.1 Instruction on how to perform the behaviour	9	5	56
5.6 Information about emotional consequences	5	3	60
13.2 Framing/reframing	5	3	60
1.2 Problem solving	32	20	63
5.1 Information about health consequences	34	22	65
1.1 Goal setting (behaviour)	20	13	65
9.1 Credible source	9	6	67
2.5 Monitoring of outcome(s) of behaviour without feedback	25	17	68
11.1 Pharmacological support	25	17	68
3.1 Social support (unspecified)	33	24	73
1.7 Review outcome goal(s)	8	6	75
1.9 Commitment	4	3	75
2.7 Feedback on outcome(s) of behaviour	8	6	75
5.4 Monitoring of emotional consequences	5	4	80
12.5 Adding objects to the environment	5	4	80
2.1 Monitoring of behaviour by others without feedback	5	5	100

^a^ ‘Index of potential’ refers to the percentage of studies found to show evidence of potential effectiveness within a BCT. Rows in green suggest BCTs found to show promise (index of potential >50%) and brown rows highlight BCTs not meeting the index of potential threshold (Gardner et al., 2017).

### Potential BCTs effectiveness

Seventeen of the 32 BCTs showed the potential for having a positive impact on primary outcomes in participants with M‐LTCs. The index of potential for all these BCTs can be seen in Table 3. The six BCTs with the highest index of potential were Monitoring of behaviour by others without feedback (IP = 100%); Monitoring of emotional consequences (IP = 80%); Adding objects to the environment (IP = 80%); Commitment (IP = 75%); Review outcome goal(s) (IP = 75%); and Feedback on outcome(s) of behaviour (75%). The other 15 BCTs were not linked with positive primary outcomes.

### Behavioural targets

Behavioural targets covered disease management (*n* = 25), nutrition (*n* = 17), physical exercise (*n* = 20), medication adherence (*n* = 25), substance use cessation (smoking, alcohol, drugs) (*n* = 10), psychological wellbeing (*n* = 21) and social inclusion (*n* = 5). Over half included interventions covering at least two behavioural targets (*n* = 31; 53%) (Table S2). Exploring the effectiveness of the intervention on the primary outcomes by the behavioural target, social inclusion (100%), substance use cessation (80%) and psychological wellbeing (62%) had the best outcomes, followed by medication adherence (60%), nutrition (59%) and physical exercise (55%), with general disease management the worst (40%).

### M‐LTCs combination type

Interventions aimed at people with both physical and mental LTCs contained more BCTs on average (7.7 vs. 6.1, respectively) and more often contained BCTs with a higher index of potential rating overall than those aimed at people with two or more physical LTCs (16 vs. 1, respectively).

Additional BCTs which are associated with psychological wellbeing were included more frequently in interventions for people with combinations of physical and mental LTCs, including 5.4 Monitoring of emotional consequences; 5.6 Information about emotional consequences; and 13.2 Framing/reframing. No specific BCTs were seen more in interventions for people with combinations of physical LTCs only.

## DISCUSSION

Across the 59 included studies, 44/93 BCTs were identified at least once, with an average of seven BCTs per intervention. Thirty‐two of these BCTs were used in three or more studies and mainly in the categories Goals and Planning, Feedback and Monitoring, and Natural Consequences.

The findings suggest that interventions for people living with M‐LTCs should include behavioural goal setting, followed by reviewing and monitoring at the outcome level. Monitoring by others without feedback was important for both the behaviour itself and the outcome from the behaviour. Feedback was most likely to be helpful when it focused on the outcome, but not for biofeedback. Self‐monitoring of both the behaviour and the outcome did not seem to result in better outcomes, suggesting that having someone else monitor and provide feedback is the key part resulting in improved outcomes. External support provided to participants was also seen in more studies with better outcomes. These included pharmacological support, adding objects to the environment to support people to complete the behaviour, and through unspecified social support from trained facilitators. However, prompts and cues were not seen to have a benefit, suggesting the support needed to be more practical. In addition, providing information about health and emotional consequences, alongside instruction on how to complete the behaviour from a credible source was also seen more in studies with favourable outcomes. However, it is important to consider that this high‐level summary does not take into account the different behavioural targets across the interventions, which may affect how beneficial a BCT is. Therefore, caution should be taken when interpreting these results.

### Results in context

These findings suggest that the most beneficial interventions for people with M‐LTCs should focus on setting a goal around the behaviour they are trying to improve, then explore potential or existing barriers to achieving this and plan ways to overcome them. Whilst this supports current recommendations for personalized care planning (Coulter et al., 2015), in clinical practice, outcome goals are set more frequently than behavioural goals due to time constraints and the practicalities of clinical practice. These findings suggest that this outdated approach needs to be addressed by training clinicians in effective goal‐setting practices, such as targeting goals at the behavioural level, to ensure patient outcomes can be improved (Légaré et al., 2008; Ørtenblad et al., 2023; Scobbie et al., 2015). One of the main challenges in implementing these findings is the need for external support to monitor behaviour and outcomes and provide feedback on outcomes in a resource‐poor system. When exploring the implementation of the NHS Health Check, it was found that the SMART goal setting was not being implemented as planned (Shaw et al., 2015). There were recommendations for more training of health care professionals around goal setting, which went beyond advice‐giving, and implementing systematic monitoring and follow‐up of these goals.

Some interventions included in this review have demonstrated how the BCTs with the potential to improve outcomes can be combined to produce positive outcomes for participants living with M‐LTCs (Katon et al., 2010). However, this is not an exhaustive list of BCTs that should or should not be included within interventions. When designing future interventions, it is important that researchers consider what the intervention is trying to change and with whom to identify which BCTs are needed (Smith et al., 2013). After determining the mechanism for action, relevant BCTs for the population and behaviour can be included, alongside or instead of the BCTs with a high index of potential (Connell et al., 2019). This reflects the findings of a similar review of physical activity and diet behaviour interventions, highlighting that problem solving and practical support were the key BCTs to promote change in this population (Lin et al., 2022).

The exploration of ‘active components’ once a trial is complete through a process evaluation is limited in the current literature (Skivington et al., 2021). Reviews exploring BCTs in people with multiple physical LTCs have found that effectiveness is correlated with the number of BCTs (Eisele et al., 2019; Hoppe et al., 2017). Unpicking which BCTs or combination of BCTs are having a beneficial effect becomes challenging and unclear. This highlights the importance of evidence‐driven, purposeful intervention design and thorough evaluation of fidelity to understand what these ‘active components’ are. This enables those developing future interventions to build on top of this knowledge and develop even more effective interventions for people living with M‐LTCs, in line with emerging recommendations to adapt or evolve existing interventions rather than starting again from scratch (Araújo‐Soares et al., 2019). When trying to implement self‐management support in primary care, no improvements in patient outcomes have been found (Kennedy et al., 2013). The authors highlighted the need for active components for self‐management support to be better understood (Kennedy et al., 2013).

Even when effective interventions, incorporating BCTs with a high index of potential, are developed, implementing them into clinical practice is very challenging in the current climate. Interviews with health care professionals about how they implement behaviour change showed that even though they understood the importance, many felt this was outside of their role and skillset (Keyworth et al., 2019). With current pressures, health care professionals felt that to be able to deliver behaviour change in routine clinical practice, their workload, environment and competence needed to be addressed (Keyworth et al., 2019). A review of reviews found similar results, with health care professionals finding most barriers relating to their capability and opportunity (Mather et al., 2022), emphasizing that training and workload are the biggest barriers that need to be addressed.

### Future research

Complex interventions for people with M‐LTCs should be developed or adapted incorporating the BCTs with a higher index of potential for them to significantly improve people's outcomes. Additional BCTs and the support needed to enact them alongside these should be considered, depending on the LTCs the participants have, the target behaviour, the context and the mechanisms for action. To implement this, intervention developers should follow existing processes for intervention development and analysis, including thorough process evaluations (Skivington et al., 2021) whilst considering the above aspects. Alongside the development of effective interventions, changes to existing services, particularly in primary care where these interventions would be delivered, are needed to ensure these results can be translated to routine clinical practice.

### Limitations

Double screening of abstracts and full texts was not possible due to capacity, which could have meant some eligible papers were excluded. Double screening was done for 10% of abstracts and 20% of full texts, with 95% independent agreement reached between the two reviewers. TR has experience screening and conducting systematic reviews in behaviour change interventions. A conservative approach to inclusion was used, and if there was any uncertainty, this was discussed with the wider team to be as systematic as possible.

Determining which BCTs are present in an intervention is dependent on the quality of the reporting of interventions within published papers. Poor reporting of complex interventions is common and could mean that not all BCTs present were captured in this synthesis. In addition, due to the nature of complex interventions, it can be difficult to disentangle the interacting components of an intervention to determine causal links between BCTs and outcomes. Exploring the interacting effects of these BCTs was beyond the scope of this review and therefore not controlled for. Finally, the behaviours targeted within these complex interventions varied greatly. Whilst we discussed effectiveness depending on the behavioural target, we did not explore how the effectiveness of interventions and the type of BCTs within an intervention varied depending on the behaviour trying to be changed.

## CONCLUSIONS

Seventeen BCTs were identified as having the potential to improve outcomes in people with M‐LTCs. These were more prevalent in interventions for those with a physical and mental LTC, in comparison to those with two or more physical LTCs. Interventions delivered to those with M‐LTCs should aim to incorporate these BCTs where appropriate and consider the mechanisms of action to identify which other BCTs should be included alongside these.

## AUTHOR CONTRIBUTIONS

**Tasmin A. Rookes:** Conceptualization; investigation; funding acquisition; writing – original draft; methodology; formal analysis; project administration. **Danielle Nimmons:** Investigation; writing – review and editing; methodology; formal analysis. **Rachael Frost:** Conceptualization; funding acquisition; writing – review and editing; methodology; supervision; investigation. **Megan Armstrong:** Writing – review and editing; supervision. **Wing Nga Tsang:** Writing – review and editing; formal analysis. **Laura Davies:** Writing – review and editing; methodology; formal analysis; project administration. **Jamie Ross:** Supervision; writing – review and editing. **Jane Hopkins:** Writing – review and editing; investigation; formal analysis. **Manoj Mistry:** Investigation; formal analysis; writing – review and editing. **Stephanie J. C. Taylor:** Writing – review and editing; supervision. **Kate Walters:** Conceptualization; investigation; funding acquisition; writing – review and editing; methodology; supervision.

## Supporting information


**Supplementary Figure 1:** Example Medline (OVID) Search Strategy for Systematic Review.

**Supplementary Figure 2:** Reference list of all the 59 included RCTs.

**Supplementary Table 1:** PRISMA 2020 checklist.

**Supplementary Table 2:** Characteristics, intervention details, findings and quality assessment of the 59 included RCTs.

**Supplementary Table 3:** Coding and summary of the behaviour change techniques used in the 59 included RCTs.

## Data Availability

The data that support the findings of this study are available from the corresponding author upon reasonable request.
