# Supplementary material for: Identifying the active components through the behaviour change techniques taxonomy in complex interventions for people living with multiple long‐term health conditions: A systematic review
Source: Br J Health Psychol. 2025 Aug 27;30(3):e70019. doi: 10.1111/bjhp.70019 (PMC12391746; doi:10.1111/bjhp.70019)
Supplement: Supplementary file 1 — Supplementary Figure 1: Example Medline (OVID) Search Strategy for Systematic Review. [file BJHP-30-0-s001.docx]

**Example Medline (OVID) Search Strategy for Systematic Review**

**Multiple long-term conditions search strategy**

Adapted from Smith et al. (2021)

1 Comorbidity/

2 (comorbid$ or co-morbid$).ti,ab.

3 (multimorbid$ or multi-morbid$).ti,ab.

4 (multidisease? or multi-disease? or (multiple adj (ill$ or disease? or condition? or syndrom$ or disorder?))).ti,ab.

5 Long term condition.ti,ab.

6 Chronic disease/

7 (multiple chronic$ adj3 (disease? or ill$ or care or condition? or disorder$ or health$ or syndrom$ or symptom$)).ti,ab.

8 ((coocur$ or co-ocur$ or coexist$ or co-exist$ or multipl$) adj3 (disease? or ill$ or care or condition? or disorder$ or health$ or medication$ or symptom$ or syndrom$)).ti,ab.

9 or/1-8

10 exp diabetes mellitus/ or diabet$.ti,ab.

11 exp hypertension/ or (hypertens$ or "high blood pressure?").ti,ab.

12 exp heart diseases/ or (((heart or cardiac or cardiovascular or coronary) adj (disease? or disorder? or failure)) or arrythmia?).ti,ab.

13 exp cerebrovascular disorders/ or ((cerebrovascular or vascular or carotoid$ or arter$) adj (disorder? or disease?)).ti,ab.

14 exp asthma/ or asthma$.ti,ab.

15 exp pulmonary disease chronic obstructive/ or (copd or (pulmonary adj2 (disease? or disorder?))).ti,ab.

16 exp hyperlipidemia/ or (hyperlipidem$ or Hypercholesterolemia$ or hypertriglyceridemia$).ti,ab.

17 exp Thyroid diseases/ or ((thyroid adj (disease? or disorder)) or hyperthyroid$ or hypothyroid$).ti,ab.

18 exp arthritis rheumatoid/ or rheumatoid arthritis.ti,ab.

19 exp mental disorders/ or (((mental or anxiety or panic or mood or psychological) adj (disease? or disorder?)) or (depression or bipolar or schizophren$ or psychos$)).ti,ab.

20 exp epilepsy/ or (epileps$ or seizure?).ti,ab.

21 exp hiv infections/ or (HIV or acquired immune$ deficiency syndrome? or (aids adj (associated or related or arteritis))).ti,ab.

22 exp neoplasms/ or (neoplasm? or cancer?).ti,ab.

23 exp kidney diseases/ or (kidney adj (disease? or disorder?)).ti,ab.

24 exp liver diseases/ or (liver adj (disease? or disorder?)).ti,ab.

25 exp osteoporosis/ or osteoporosis.ti,ab.

26 exp dementia/ or dementia.ab,ti.

27 exp stroke/ or stroke.ab,ti.

28 exp multiple sclerosis/ or (multiple sclerosis).ab,ti.

29 exp Parkinson disease/ or Parkinson*.ab,ti.

30 exp motor neuron disease/ or (motor neuron disease).ab,ti.

31 exp neurodegenerative diseases/ or neurodegenerative.ab,ti.

32 or/10-31

33 9 and 32

**Behaviour change intervention search strategy**

Adapted from Gardener et al. (2017)

1 (self car*) OR (self-manag*).mp.

2 behavio* adj3 chang*.mp.

3 (health education) OR (structure* education).mp.

4 secondary prevent*.mp.

5 (case manag*) OR (care manag*).mp.

6 (care navigat*) OR (community navigat*) OR (care co-ordinat*).mp.

7 (shared care) OR (collaborative care).mp.

8 (complex intervention*) OR (multicomponent intervention) OR (multiple intervention component).mp.

9 (integrated adj (model OR care)).mp.

10 ((Multiprofessional AND intervention$) OR (Interprofessional AND intervention$)).mp.

11 (comprehensive care) OR (comprehensive health care).mp.

12 (prevent* adj3 hospital*).mp.

13 or/1-12

**RCT search strategy**

Cochrane RCT from Smith et al. (2021)

1 randomi?ed controlled trial.pt.

2 controlled clinical trial.pt.

3 random$.ti,ab.

4 (control$ adj2 (trial? or study or studies)).ti,ab.

5 double-blind method/ or random allocation/ or single-blind method/

6 ((double or single or triple or treble) adj2 blind$).ti,ab.

7 or/1-6

8 exp animals/ not humans.sh.

9 7 not 8
