# Supplementary material for: Identifying the active components through the behaviour change techniques taxonomy in complex interventions for people living with multiple long‐term health conditions: A systematic review
Source: Br J Health Psychol. 2025 Aug 27;30(3):e70019. doi: 10.1111/bjhp.70019 (PMC12391746; doi:10.1111/bjhp.70019)
Supplement: Supplementary file 2 — Supplementary Figure 2: Reference list of all the 59 included RCTs. [file BJHP-30-0-s002.docx]

1. Ali MK, Chwastiak L, Poongothai S, Emmert-Fees KMF, Patel SA, Anjana RM, et al. Effect of a Collaborative Care Model on Depressive Symptoms and Glycated Hemoglobin, Blood Pressure, and Serum Cholesterol among Patients with Depression and Diabetes in India: The INDEPENDENT Randomized Clinical Trial. JAMA - Journal of the American Medical Association. 2020 Aug 18;324(7):651–62.

2. Aragonès E, Rambla C, López-Cortacans G, Tomé-Pires C, Sánchez-Rodríguez E, Caballero A, et al. Effectiveness of a collaborative care intervention for managing major depression and chronic musculoskeletal pain in primary care: A cluster-randomised controlled trial. J Affect Disord. 2019 Jun 1;252:221–9.

3. Barley EA, Walters P, Haddad M, Phillips R, Achilla E, McCrone P, et al. The UPBEAT nurse-delivered personalized care intervention for people with coronary heart disease who report current chest pain and depression: A randomised controlled pilot study. PLoS One. 2014 Jun 5;9(6).

4. Barrowclough C, Haddock G, Tarrier N, Shôn Lewis FbpW, Jan Moring Frcp, Nichola Schofield B, et al. Article Randomized Controlled Trial of Motivational Interviewing, Cognitive Behavior Therapy, and Family Intervention for Patients With Comorbid Schizophrenia and Substance Use Disorders. Vol. 158, Am J Psychiatry. 2001.

5. Battersby MW, Beattie J, Pols RG, Smith DP, Condon J, Blunden S. A randomised controlled trial of the Flinders ProgramTM of chronic condition management in Vietnam veterans with co-morbid alcohol misuse, and psychiatric and medical conditions. Australian and New Zealand Journal of Psychiatry. 2013 May;47(5):451–62.

6. Blank MB, Hanrahan NP, Fishbein M, Wu ES, Tennille JA, Ten Have TR, et al. A randomized trial of a nursing intervention for HIV disease management among persons with serious mental illness. Psychiatric Services. 2011 Nov 1;62(11):1318–24.

7. Boeschoten RE, Dekker J, Uitdehaag BMJ, Beekman ATF, Hoogendoorn AW, Collette EH, et al. Internet-based treatment for depression in multiple sclerosis: A randomized controlled trial. Multiple Sclerosis. 2017 Jul 1;23(8):1112–22.

8. Bogner HR, De Vries HF. Integration of depression and hypertension treatment: A pilot, randomized controlled trial. Ann Fam Med. 2008;6(4):295–301.

9. Chen S, Conwell Y, Xue J, Li L, Zhao T, Tang W, Bogner H, Dong H. Effectiveness of integrated care for older adults with depression and hypertension in rural China: A cluster randomized controlled trial. PLoS medicine. 2022 Oct 24;19(10):e1004019.

10. Chwastiak LA, Luongo M, Russo J, Johnson L, Lowe JM, Hoffman G, et al. Use of a mental health center collaborative care team to improve diabetes care and outcomes for patients with psychosis. Psychiatric Services. 2018 Mar 1;69(3):349–52.

11. Cummings DM, Lutes LD, Littlewood K, Solar C, Carraway M, Kirian K, et al. Randomized trial of a tailored cognitive behavioral intervention in type 2 diabetes with comorbid depressive and/or regimen-related distress symptoms: 12-month outcomes from. In: Diabetes Care. American Diabetes Association Inc.; 2019. p. 841–8.

12. Druss BG, Singh M, Von Esenwein SA, Glick GE, Tapscott S, Tucker SJ, et al. Peer-led self-management of general medical conditions for patients with serious mental illnesses: A randomized trial. Psychiatric Services. 2018 May 1;69(5):529–35.

13. Ell K, Katon W, Xie B, Lee PJ, Kapetanovic S, Guterman J, et al. Collaborative care management of major depression among low-income, predominantly hispanic subjects with diabetes: A randomized controlled trial. Diabetes Care. 2010 Apr;33(4):706–13.

14. Ell K, Aranda MP, Wu S, Oh H, Lee PJ, Guterman J. Promotora assisted depression and self-care management among predominantly Latinos with concurrent chronic illness: Safety net care system clinical trial results. Contemp Clin Trials. 2017 Oct 1;61:1–9.

15. Fisher K, Markle-Reid M, Ploeg J, Bartholomew A, Griffith LE, Gafni A, et al. Self-management program versus usual care for community-dwelling older adults with multimorbidity: A pragmatic randomized controlled trial in Ontario, Canada. J Comorb. 2020 Jan 1;10:2235042X2096339.

16. Garvey J, Connolly D, Boland F, Smith SM. OPTIMAL, an occupational therapy led self-management support programme for people with multimorbidity in primary care: A randomized controlled trial. BMC Fam Pract. 2015 Dec 12;16(1).

17. Goldberg RW, Dickerson F, Lucksted A, Brown CH, Weber E, Tenhula WN, et al. Living Well: An intervention to improve self-management of medical illness for individuals with serious mental illness. Psychiatric Services. 2013 Jan 1;64(1):51–7.

18. Goorden M, van der Feltz-Cornelis CM, van Steenbergen-Weijenburg KM, Horn EK, Beekman ATF, Hakkaar-Van Roijen L. Cost-utility of collaborative care for the treatment of comorbid major depressive disorder in outpatients with chronic physical conditions. A randomized controlled trial in the general hospital setting (CC-DIM). Neuropsychiatr Dis Treat. 2017 Jul 18;13:1881–93.

19. Griva K, Rajeswari M, Nandakumar M, Khoo EYH, Lee VYW, Chua CG, et al. The combined diabetes and renal control trial (C-DIRECT) - A feasibility randomised controlled trial to evaluate outcomes in multi-morbid patients with diabetes and on dialysis using a mixed methods approach. BMC Nephrol. 2019 Jan 3;20(1).

20. Guo Y, Lane DA, Wang L, Zhang H, Wang H, Zhang W, et al. Mobile Health Technology to Improve Care for Patients With Atrial Fibrillation. J Am Coll Cardiol. 2020 Apr 7;75(13):1523–34.

21. Hernandez-Quiles C, Bernabeu-Wittel M, Barón-Franco B, Palacios AA, Garcia-Serrano MR, Lopez-Jimeno W, et al. A randomized clinical trial of home telemonitoring in patients with advanced heart and lung diseases. J Telemed Telecare. 2021;

22. House A, Bryant L, Russell AM, Wright-Hughes A, Graham L, Walwyn R, et al. Randomized controlled feasibility trial of supported self-management in adults with Type 2 diabetes mellitus and an intellectual disability: OK Diabetes. Diabetic Medicine. 2018 Jun 1;35(6):776–88.

23. Hoyo ML, Rodrigo MT, Urcola-Pardo F, Monreal-Bartolomé A, Ruiz DC, Borao MG, Alcázar AB, Casbas JP, Casas AA, Funcia MT, Delgado JF. The TELE-DD randomised controlled trial on treatment adherence in patients with type 2 diabetes and comorbid depression: clinical outcomes after 18-month follow-up. International Journal of Environmental Research and Public Health. 2022 Dec 25;20(1):328.

24. Jackson IL, Ukwe C V. Clinical outcomes of pharmaceutical care intervention in HIV positive patients with hypertension: A randomized controlled study. J Clin Pharm Ther. 2021 Aug 1;46(4):1083–94.

25. Kamradt M, Ose D, Krisam J, Jacke C, Salize HJ, Besier W, et al. Meeting the needs of multimorbid patients with Type 2 diabetes mellitus – A randomized controlled trial to assess the impact of a care management intervention aiming to improve self-care. In: Diabetes Research and Clinical Practice. Elsevier Ireland Ltd; 2019. p. 184–93.

26. Kanwal F, Pyne JM, Tavakoli-Tabasi S, Nicholson S, Dieckgraefe B, Storay E, et al. A Randomized Trial of Off-Site Collaborative Care for Depression in Chronic Hepatitis C Virus. Health Serv Res. 2018 Aug 1;53(4):2547–66.

27. Katon WJ, Michael ;, Korff V, Lin EHB, Simon G, Ludman E, et al. The Pathways Study A Randomized Trial of Collaborative Care in Patients With Diabetes and Depression. 2004.

28. Katon WJ, Lin EH, Von Korff M, Ciechanowski P, Ludman EJ, Young B, et al. Collaborative Care for Patients with Depression and Chronic Illnesses From the Departments of Psychiatry A bs t r ac t. Vol. 363, N Engl J Med. 2010.

29. Khunti K, Highton PJ, Waheed G, Dallosso H, Redman E, Batt ME, et al. Promoting physical activity with self-management support for those with multimorbidity: A randomised controlled trial. British Journal of General Practice. 2021 Dec 1;71(713):E921–30.

30. Koesoemadinata RC, McAllister SM, Soetedjo NNM, Santoso P, Ruslami R, Damayanti H, et al. Educational counselling of patients with combined TB and diabetes mellitus: a randomised trial. Public Health Action. 2021 Dec 18;11(4):202–8.

31. Kroenke K, Bair MJ, Damush TM, Wu J, Hoke S, Sutherland J, et al. Optimized Antidepressant Therapy and Pain Self-management in Primary Care Patients With Depression and Musculoskeletal Pain A Randomized Controlled Trial [Internet]. 2009. Available from: https://jamanetwork.com/

32. Lear SA, Norena M, Banner D, Whitehurst DGT, Gill S, Burns J, et al. Assessment of an Interactive Digital Health-Based Self-management Program to Reduce Hospitalizations among Patients with Multiple Chronic Diseases: A Randomized Clinical Trial. JAMA Netw Open. 2021 Dec 28;4(12).

33. Lenferink A, van der Palen J, van der Valk PDLPM, Cafarella P, van Veen A, Quinn S, et al. Exacerbation action plans for patients with COPD and comorbidities: A randomised controlled trial. European Respiratory Journal. 2019 Nov 1;54(5).

34. Markle-Reid M, Ploeg J, Fraser KD, Fisher KA, Bartholomew A, Griffith LE, et al. Community Program Improves Quality of Life and Self-Management in Older Adults with Diabetes Mellitus and Comorbidity. J Am Geriatr Soc. 2018 Feb 1;66(2):263–73.

35. Markle-Reid M, Fisher K, Walker KM, Beauchamp M, Cameron JI, Dayler D, Fleck R, Gafni A, Ganann R, Hajas K, Koetsier B. The stroke transitional care intervention for older adults with stroke and multimorbidity: a multisite pragmatic randomized controlled trial. BMC geriatrics. 2023 Oct 24;23(1):687.

36. McBain RK, Mwale O, Mpinga K, Kamwiyo M, Kayira W, Ruderman T, Connolly E, Watson SI, Wroe EB, Munyaneza F, Dullie L. Effectiveness, cost-effectiveness, and positive externalities of integrated chronic care for adults with major depressive disorder in Malawi (IC3D): a stepped-wedge, cluster-randomised, controlled trial. The Lancet. 2024 Nov 9;404(10465):1823-34.

37. McDermott RA, Schmidt B, Preece C, Owens V, Taylor S, Li M, et al. Community health workers improve diabetes care in remote Australian indigenous communities: Results of a pragmatic cluster randomized controlled trial. BMC Health Serv Res. 2015 Feb 19;15(1).

38. Merlin JS, Westfall AO, Long D, Davies S, Saag M, Demonte W, et al. A Randomized Pilot Trial of a Novel Behavioral Intervention for Chronic Pain Tailored to Individuals with HIV. AIDS Behav. 2018 Aug 1;22(8):2733–42.

39. Miklavcic JJ, Fraser KD, Ploeg J, Markle-Reid M, Fisher K, Gafni A, et al. Effectiveness of a community program for older adults with type 2 diabetes and multimorbidity: A pragmatic randomized controlled trial. BMC Geriatr. 2020 May 13;20(1).

40. Morgan MAJ, Coates MJ, Dunbar JA, Reddy P, Schlicht K, Fuller J. The TrueBlue model of collaborative care using practice nurses as case managers for depression alongside diabetes or heart disease: a randomised trial. BMJ Open [Internet]. 2013; Available from: http://bmjopen.bmj.com/

41. Naik AD, Hundt NE, Vaughan EM, Petersen NJ, Zeno D, Kunik ME, et al. Effect of Telephone-Delivered Collaborative Goal Setting and Behavioral Activation vs Enhanced Usual Care for Depression among Adults with Uncontrolled Diabetes: A Randomized Clinical Trial. JAMA Netw Open. 2019 Aug 7;2(8).

42. Ose D, Kamradt M, Kiel M, Freund T, Besier W, Mayer M, et al. Care management intervention to strengthen self-care of multimorbid patients with type 2 diabetes in a German primary care network: A randomized controlled trial. PLoS One. 2019 Jun 1;14(6).

43. O’Toole L, Connolly D, Boland F, Smith SM. Effect of the OPTIMAL programme on self-management of multimorbidity in primary care: A randomised controlled trial. British Journal of General Practice. 2021 Apr 1;71(705):E303–11.

44. Park YH, Chang HK. Effect of a health coaching self-management program for older adults with multimorbidity in nursing homes. Patient Prefer Adherence. 2014 Jul 4;8:959–70.

45. Pibernik-Okanovic M, Begic D, Ajdukovic D, Andrijasevic N, Metelko Z. Psychoeducation versus treatment as usual in diabetic patients with subthreshold depression: Preliminary results of a randomized controlled trial. Trials. 2009 Aug 26;10.

46. Rose L, Istanboulian L, Carriere L, Thomas A, Lee HB, Rezaie S, et al. Program of integrated care for patients with chronic obstructive pulmonary disease and multiple comorbidities (PIC COPD+): A randomised controlled trial. European Respiratory Journal. 2018;51(1).

47. Ross CJM, Davis TMA, MacDonald GF. Cognitive-behavioral treatment combined with asthma education for adults with asthma and coexisting panic disorder. Clin Nurs Res. 2005 May;14(2):131–57.

48. Sajatovic M, Gunzler DD, Kanuch SW, Cassidy KA, Tatsuoka C, McCormick R, et al. A 60-week prospective RCT of a self-management intervention for individuals with serious mental illness and diabetes mellitus. Psychiatric Services. 2017 Sep 1;68(9):883–90.

49. Salisbury C, Man MS, Bower P, Guthrie B, Chaplin K, Gaunt DM, et al. Management of multimorbidity using a patient-centred care model: a pragmatic cluster-randomised trial of the 3D approach. The Lancet. 2018 Jul 7;392(10141):41–50.

50. Schneider KL, Panza E, Handschin B, Ma Y, Busch AM, Waring ME, et al. Feasibility of Pairing Behavioral Activation With Exercise for Women With Type 2 Diabetes and Depression: The Get It Study Pilot Randomized Controlled Trial [Internet]. 2015. Available from: www.elsevier.com/locate/bt

51. Srinivasan K, Heylen E, Johnson Pradeep R, Mony PK, Ekstrand ML. Collaborative care compared to enhanced standard treatment of depression with co-morbid medical conditions among patients from rural South India: a cluster randomized controlled trial (HOPE Study). Bmc Psychiatry. 2022 Jun 13;22(1):394.

52. Takahashi PY, Pecina JL, Upatising B, Chaudhry R, Shah ND, Van Houten H, et al. A randomized controlled trial of telemonitoring in older adults with multiple health issues to prevent hospitalizations and emergency department visits. Arch Intern Med. 2012 May 28;172(10):773–9.

53. Taylor BC, Houston Miller N, Reilly KR, Greenwald G, Cunning D, Deeter A, et al. Evaluation of a Nurse-Care Management System to Improve Outcomes in Patients With Complicated Diabetes [Internet]. 2003. Available from: http://diabetesjournals.org/care/article-pdf/26/4/1058/659413/dc0403001058.pdf

54. Vera M, Perez-Pedrogo C, Huertas SE, Reyes-Rabanillo ML, Juarbe D, Huertas A, et al. Collaborative Care for Depressed Patients With Chronic Medical Conditions: A Randomized Trial in Puerto Rico. Psychiatric Services. 2010 Feb 1;61(2).

55. Wakefield BJ, Holman JE, Ray A, Scherubel M, Adams MR, Hillis SL, et al. Effectiveness of home telehealth in comorbid diabetes and hypertension: A randomized, controlled trial. Telemedicine and e-Health. 2011 May 1;17(4):254–61.

56. Wang Y, Guo D, Xia Y, Hu M, Wang M, Yu Q, Li Z, Zhang X, Ding R, Zhao M, Shi Z. Effect of Community-Based Integrated Care for Patients With Diabetes and Depression (CIC-PDD) in China: A Pragmatic Cluster-Randomized Trial. Diabetes Care. 2025 Feb 1;48(2):226-34.

57. Williams A, Manias E, Walker R, Gorelik A. A multifactorial intervention to improve blood pressure control in co-existing diabetes and kidney disease: A feasibility randomized controlled trial. J Adv Nurs. 2012 Nov;68(11):2515–25.

58. Wilson M, Hewes C, Barbosa-Leiker C, Mason A, Wuestney KA, Shuen JA, et al. Engaging Adults With Chronic Disease in Online Depressive Symptom Self-Management. West J Nurs Res. 2018 Jun 1;40(6):834–53.

59. Yu C, Choi D, Bruno BA, Thorpe KE, Straus SE, Cantarutti P, et al. Impact of mydiabetesplan, a web-based patient decision aid on decisional conflict, diabetes distress, quality of life, and chronic illness care in patients with diabetes: Cluster randomized controlled trial. J Med Internet Res. 2020 Sep 29;22(9).
